# Magnitude and pattern of hypertension in the Niger Delta: a systematic review and meta-analysis of community-based studies

**DOI:** 10.7189/jogh.08.010420

**Published:** 2018-06

**Authors:** Martinsixtus Ezejimofor, Olalekan Uthman, Yen-Fu Chen, Benedeth Ezejimofor, Aloysius Ezeabasili, Saverio Stranges, Ngianga-Bakwin Kandala

**Affiliations:** 1 Division of Health Sciences, University of Warwick Medical School, Coventry, UK; 2 British Association of Dermatologists, Willan House, Fitzroy Square, London, UK; 3 Warwick-Centre for Applied Health Research and Delivery, Division of health Sciences, University of Warwick Medical School, Coventry, UK; 4 School of the Built Environment, University of Salford, Salford, Greater Manchester, UK; 5 Department of Epidemiology and Biostatistics, Schulich School of Medicine & Dentistry, Western University, London, Ontario, Canada; 6 Department of Population Health, Luxembourg Institute of Health, Strassen, Luxembourg; 7 Northumbria University, Department of Mathematics and Information sciences, Faculty of Engineering and Environment, Newcastle upon Tyne, United Kingdom.; 8 University of the Witwatersrand, Division of Epidemiology and Biostatistics, School of Public Health, Johannesburg, South Africa

## Abstract

**Background:**

Emerging evidence found that health inequality in the Niger Delta region in Nigeria has continued to worsen due to epidemiological and environmental risks transitions. This study aims to provide an up-to-date review and the secular trends of hypertension prevalence in Niger Delta.

**Methods:**

We systematically searched databases of MEDLINE, EMBASE, African index Medicus and African Journal online from inception to December 30, 2016 for population-based studies providing prevalence estimates of hypertension in the Niger Delta. Eligible studies were included in a random-effect meta-analysis of prevalence and secular trend. The review was reported according to MOOSE guideline.

**Results:**

Overall, 34 eligible studies comprising of data on 32715 participants with mean-age of 38.43 ± 2.0 years were identified and included in the meta-analysis. The pooled result showed that across study settings, the prevalence of hypertension in rural population tended to be higher than those in urban areas, 32.0% (95% confidence interval (CI) 25.13-39.28) vs 24.07% (95% CI 18.13-30.58), however, the difference did not reach a statistical significant level, (*P* < 0.183). The overall mean SBP was 130.15 (95% CI 126.85-133.45) mmHg, and the DBP was 80.72 (95% CI 78.45-82.95). The estimates also vary significantly in men compared to women; 30.26% (95% CI 23.76-37.17) vs 22.99% (17.60-28.86), *P* < 0.0001, and among those older than 65 years compared to those aged 45-64 years, and more than 2-fold compared to those between 15-44 years, *P* < 0.001. We also observed a continuous increase in prevalence of hypertension in the region (trend = 0.139, *P* = 0.0001), such that for every 10 years increase in participants’ mean age, the prevalence of hypertension increases by 10.43% (95% CI 5.73-15.14), *P* < 0.001.

**Conclusions:**

This study found evidence that hypertension is a major public health issue in the Niger Delta communities suggesting a positive relationship between socio-economic and lifestyle factors. Improved surveillance and care, as well as better management of the underlying risk factors, primarily undetected or uncontrolled high blood pressure, remains an important public health priority.

Hypertension has continued to drive the global burden of cardiovascular disease as the most common cardiovascular disorder and number one risk factor for mortality [1]. Recent estimate revealed that nearly one billion people have hypertension globally; of these, two-thirds are in developing countries [1]. In real terms, about 640 million people have hypertension in low- and middle-income countries (LMICs). This is a huge contrast to 330 million people in high-income countries. However, this number is expected to increase to 1.56 billion adults living with hypertension in 2025 with more than two-third occurring in LMIC [2].The increase in the burden of hypertension in LMICs has been attributed to both intrinsic and extrinsic factors [2]. In particular, the ongoing epidemiological transition driven by nutritional and demographic changes, increasing trends in sedentary lifestyle and other modifiable risk factors and inadequate health systems have been some major contributory factors [3,4].

In sub-Saharan Africa (SSA) particularly Nigeria, evidence of double burden of non-communicable diseases (such as stroke and hypertension and injuries), and communicable diseases such as HIV/AIDS, Malaria and other vaccine preventable diseases in the face of chronic poverty and hunger dominates the public health landscape [5,6]. While infectious diseases have received huge attention given the near-success of the Millennium Development Goals (MDGs), non-communicable disease particularly hypertension has continued to mount. Recent evidence revealed that Nigeria and many other countries in SSA is at the second stage of the epidemiological transition characterised by hypertensive heart disease driven by cardiovascular risk factors [7]. The surge in urbanisation and industrialisation in these countries have made modification of dietary consumption including increases in dietary salt and food rich in bad cholesterol inevitable. In addition to these, environmental risk exposures appear to be occurring simultaneously. Direct research evidence linking these exposures to relevant public health conditions appear to have been underreported in the Niger Delta due to inadequate research funding; however, it is not in doubt that several decades of oil and gas exploration and exploitation may have had serious environmental impact including poverty, malnutrition and chronic health condition leading to reduced life expectancy [8,9].

Recent reviews and meta-analysis evidence of the prevalence of hypertension in Nigeria have reported huge estimates and significant variation ranging from 8.9 to 46% [10,11]. The review also found that the burden of hypertension has been highest in urban areas compared to rural environment with values of 30.6% vs 26.4% respectively [10]. These reviews reported the influence of lifestyle factors as one of the major risk factors to the significantly higher hypertension burden in urban areas compared to the rural settings. This evidence is consistent with the finding of many reviews which support the current transitions in sub-Saharan Africa and many LMICs that started to manifest few decades ago [12,13].

Industrialisation and urbanisation in the Niger Delta in Nigeria have given rise to rapid nutritional transition towards consumption of ready-to-eat food with high salt, sugar and fat contents, replacing unprocessed natural products that are still common in rural communities [11,14-16]. These nutritional changes are also spreading rapidly in the rural oil and gas host communities in the Niger Delta. In addition to diets, urban and rural living in the region also encourage high alcohol consumption and smoking, and sedentary lifestyles through the acquisition of new technology, increased office jobs, conversion of agricultural lands to oil and gas facilities and motorised transportation that make physical activity more difficult [2,11,17]. These exposures could be linked to increased risk of hypertension and other metabolic conditions such as obesity and diabetes.

With the adoption of unhealthy lifestyle brought about by ongoing urbanisation and increased oil and gas production activities, we argued that the available evidence on the burden of hypertension and comorbidities might have been a misrepresentation of the true situation in the Niger Delta. This review aims to estimate the prevalence of hypertension among adults (15 years and over) and examine secular trends, geographic and socioeconomic variations in Niger Delta. The evidence could stimulate increased attention and initiatives by health policy makers to mitigate this emerging and potential public health issue not only in the Niger Delta but also nationally.

## METHODS

### Search strategy

A thorough literature search was conducted to identify relevant studies on hypertension prevalence in the Niger Delta in Nigeria. Electronic databases of Medline, Embase, African index Medicus and African Journal online were systematically searched from inception to December 30, 2016 without any language restriction. Relevant articles were identified using the following combinations of MeSH controlled terms, keywords and boolean operators. These include; hypertension” OR “blood pressure” OR “hypertens*” and “surveillance” OR “survey” OR “prevalence” OR “burden” OR “population-base” OR “community-based” OR “etiology” OR “aetiology” OR “epidemiolog*” and “Niger Delta”; including all the individual States” OR “oil producing communit*”. Reference lists of eligible articles were also scrutinized for additional studies that could have been omitted from the database searches. Additionally, authors of selected articles were also contacted to provide specific or missing information regarding their studies and any other published or unpublished work.

### Selection criteria

Retrieved articles were initially screened by their titles and abstracts to obtain studies that met the following selection criteria. These include; a population and/or community-based studies conducted from inception to December 30, 2016. We considered studies that recruited participants aged 15 years and over living in any of the States in the Niger Delta, Nigeria. Such study must report the prevalence of hypertension or provide numerical estimates from which the prevalence of hypertension could be estimated. We included all studies in which hypertension was defined based on 140/90, 160/95 and 160/100 mm Hg [18-20]. Studies were excluded if they recruited pregnant women or participants below 15 years of age. Equally excluded were studies without clearly defined diagnostic criteria and blood pressure measurement protocols. Hospital-based studies, policy report or reviews, and studies that did not contain original data (primary data) or focused on non-humans were also excluded. We considered studies based on the current and previously accepted definitions of hypertension, (systolic blood pressure of 140-160 mm Hg or a diastolic blood pressure of 90-100 mm Hg), based on recommendations of the Joint National Committee on Prevention, Detection, Evaluation, and Treatment of High Blood Pressure [18-20]. We also adopted studies with a subjective definition of hypertension based on physician-diagnosed hypertension or use of antihypertensive medication due to elevated systolic or diastolic blood pressure measurement.

### Study selection procedure

Appropriate studies included in the review were obtained through clearly defined stages of selection process. First, the titles and abstracts of the articles obtained through the search were screened for relevance by two independent authors (ME and AE). Any disagreement was resolved through consensus. In the second stage, full texts of the selected articles were retrieved. Reference list of the selected articles were also scanned for additional publications. All the retrieved articles were read for compliance based on the established selection criteria. In the final stage, only studies that met the selection criteria were included in the quality and risk assessment evaluation.

### Quality assessment and risk of bias

Three authors (ME, AE and BE) independently evaluated the methodological and reporting quality of the individual studies and transcripts using the modified version of Newcastle-Ottawa Scale [21]. In cases of discrepancy, agreement was reached by consensus. We used funnels plot and Egger’s test to assess publication bias for the small study effect [22].

### Data extraction

For each included study, data and other details were abstracted systematically using a standardised protocol and piloted online data extraction form. Relevant study information extracted includes author’s name, publication date, study period, publication type, State, sampling procedure, gender proportion, study setting, age-range, mean-age, crude prevalence, and sample size, diagnostic criteria/case ascertainment, confounders, comorbidities, analytic method, limitations and key finding from each of the studies.

### Statistical analysis

The overall prevalence of hypertension was pooled and compared across study settings using a meta-analysis. Before this, we first stabilized the raw proportions of subjects with hypertension from each of the included study using the Freeman-Tukey variant of the arcsine square root transformed proportion suitable for pooling [23]. Thereafter, the DerSimonian and Laird random effects model was used to summarize the data [24]. We assessed study variations (within- and between-studies variability) by inspecting the forest plot and by using the *I^2^* statistics (we interpret a value of 50% as representing moderate heterogeneity) and the χ^2^ test, to test subgroup differences. The result presented as a forest plots with 95% confidence intervals (CIs) expressed in percentage. The prevalence of hypertension was also evaluated using study-level data on participant characteristics (age group, gender, alcohol use and smoking status).

We also examined the time trends in the prevalence estimates of hypertension from 1980 to 2016 using Poisson regression models with the absolute cases of hypertension as the outcome variable and the calendar year of the publication as the predictor. This method allows for estimation of time trends across individual calendar years to obtain average annual percentage change (AAPC), assuming that the rate of change is at a constant rate of the previous year [25]. We used a significance level of 0.05 for *P*-values in all statistical analysis. All data analysis was conducted using Stata version 14 for Windows (Stata Corp, College Station, Texas). This study was conducted and reported in line with the Meta-analysis of Observational Studies in Epidemiology (MOOSE) guideline [26].

## RESULTS

### Search strategy results

**Figure 1:**
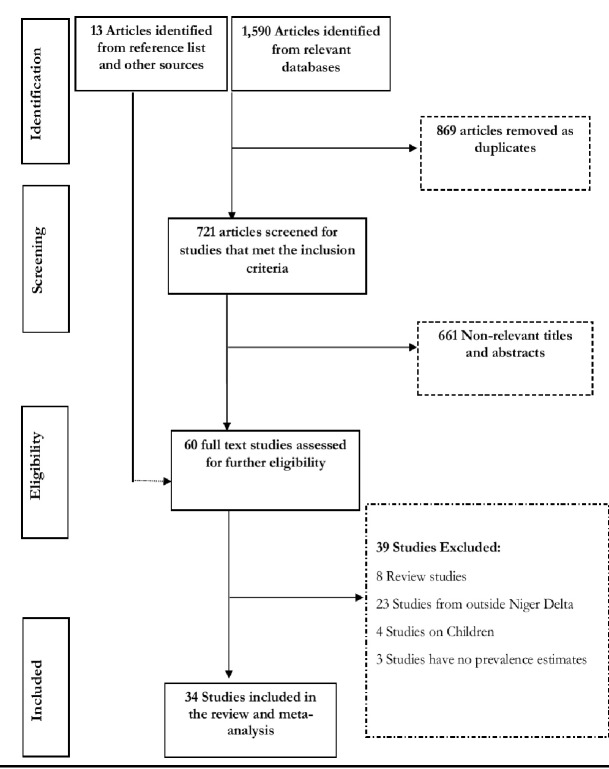
Flow diagram of search results.

**Figure 1** shows the flow diagram of search results. The literature search of databases returned 1590 publications. After removing duplicates and following the screening of the titles and abstracts of the publications, we selected 60 articles with full texts for critical reading. A further 39 articles were excluded (24 were conducted outside the Niger Delta, 8 were review articles, 4 were among children and 3 have no hypertension prevalence estimates). We also obtained 13 relevant articles from the reference list of the selected studies. In all, 34 relevant and quality studies conducted in 8 states (Bayelsa, Akwa Ibom, Cross River, Delta, Edo, Rivers, Abia, Imo) that satisfied our selection criteria were obtained and included in the meta-analysis.

### Study characteristics

The characteristics of the 34 included studies are shown in **Table 1**. The breakdown of the included studies shows that that Edo State has the highest number (n = 8) of studies while Bayelsa State has 2 studies only. We collated data from 34 included studies from 8 out of 9 States that comprise the Niger Delta where about 32 million people currently live (according to 2006 Nigerian census and with 2.9% population growth rate in 2017). All the studies were cross-sectional population or community-based studies employing a door-to door, multi-stage cluster or simple random sampling technique in the recruitment of participants. The included studies had a total sample size of 32715 including 19931 men (60.92%) and 12784 women (39.08%). Two studies have hypertension cut-off points of 160/95 and 160/100 mm Hg [27,28] while 31 studies used a cut-off of 140/90mm Hg (Table 1). We found that 17 studies (50.0%) were conducted in predominantly urban settings, while 12 (35.29%) and 5 (14.71%) studies were conducted in rural and rural/urban settings respectively. All the studies had overall mean-age of 39.93 ± 2.0 years with values of 41.21 ± 10.8 in rural, 37.47 ± 7.24 in urban settings and 41.50 ± 7.70 in rural/urban settings.

**Table 1:** Characteristics of selected hypertension studies in the Niger Delta

First Author	Data Collection year	State	Setting	Age	Mean age	Sample size	Male percentage	Hypertension prevalence	Diagnostic Criteria (BP cut-off)	Quality Grading
Oviasu [27]	NR	Edo	Urban	15-60	NR	1263	NR	13.3	160/95	Moderate
Idahosa[28]	1983	Edo	Urban	15-70	31.8	1450	100	11.6	160/100	Moderate
Idahosa [29]	1983	Edo	Urban	20-62	27.8	1115	NR	28.7	140/90	Moderate
Bunker[30]	1987-88	Cross River	Urban	25-54	36.4	559	78.35	30.41	140/90	Moderate
Okojie [31]	NR	Edo	Urban	25-64	NR	202	76.7	34.65	140/90	Moderate
Omuemu [32]	NR	Edo	Rural	≥15	30.7 ± 14.6	590	60.2	20.2	140/90	Moderate
Ofuya [33]	NR	Rivers	Rural	16-56	23.3	200	NR	14	140/90	Moderate
Ike [34]	2004	Abia	Urban	21-70	43.7 ± 10	85	91.7	28.3	140/90	Moderate
Akpa [35]	NR	Rivers	Urban/Rural	≥18	39.9 ± 8.6	921	48.75	40.82	140/90	Moderate
Omorogiuwa [36]	NR	Edo	Urban	≥18	NR	1200	40	33	140/90	Moderate
Onwuchekwa [14]	2008	Rivers	Rural	≥18	35.8 ± 14.8	1078	44	18.3	140/90	High
Andy [37]	2012	Cross River	Rural	≥15	34.1 ± 14.4	3869	41.6	23.6	140/90	Moderate
Ekpenyon[6]	2009-10	Akwa Ibom	Urban	18-60	NR	2780	52	14.4	140/90	Moderate
Suleiman [38]	2011	Bayelsa	Urban	≤20	NR	400	40	15	140/90	Moderate
Ordinioha [39]	NR	Rivers	Urban/Rural	15-60	46.1 ± 10	75	65.33	21.33	140/90	Moderate
Okpechi [40]	2011-12	Abia	Urban/Rural	≥18	41.7	2983	47.9	31.4	140/90	Moderate
Ordinioha[16]	2012	Rivers	Rural	≥18	56.5 ± 4.1	106	100	68.86	140/90	Moderate
Ijezie [41]	NR	Abia	Urban/Rural	≥18	41.8 ± 18.7	2807	49.09	36.59	140/90	High
Ekanem [42]	2010	Akwa Ibom	Urban	16-64	31.7 ± 7.6	444	51.6	47	140/90	Moderate
Mbah[43]	NR	Imo	Rural	40-60	NR	200	40	35.5	140/90	Moderate
Ganiyu [44]	2013	Delta	Rural	≥25	39.2 ± 12.1	500	51.8	29.8	140/90	High
Egbi [45]	2013	Bayelsa	Urban	≥18	37.2 ± 8.9	231	36.4	21.3	140/90	High
Oguoma[46]	2014	Delta	Urban/Rural	≥18	51.3 ± 2	145	38.6	37.3	140/90	Moderate
Oguoma[46]	2014	Delta	Urban/Rural	≥18	28.2 ± 1	181	28.2	23.2	140/90	Moderate
Isara[47]	2013	Edo	Rural	≥18	NR	845	31.12	37.8	140/90	Moderate
Ibekwe[48]	2012	Delta	Urban	≥18	36.7 ± 14	272	51.1	21	140/90	High
Odili [49]	2012	Imo	Rural	≥18	54.7 ± 16.3	122	45.1	54.1	140/90	Moderate
Alikor [50]	NR	Rivers	Rural	≥18	41.32	500	31.2	20.2	140/90	Moderate
Okwuonu [51]	2013	Abia	Urban	≥18	52.1	389	57.3	37.8	140/90	Moderate
Onoh [52]	NR	Imo	Urban	15-64	NR	107	51.4	48.6	140/90	Moderate
Akpan [53]	2013	Akwa Ibom	Urban	≥18	39.9	590	26.3	28.6	140/90	High
Akpan [53]	2013	Akwa Ibom	Rural	≥18	43.9	978	29.9	44.3	140/90	High
Ofili [54]	2014	Delta	Rural	≥20	52.6	134	35.8	44	140/90	Moderate
Diwe [55]	NR	Imo	Urban	≥20	NR	194	51.5	12.5	140/90	Moderate
Ekpo [56]	1992	Cross River	Urban	≥18	NR	4382	84.2	8.1	140/90	Moderate

NR – not reported

### Result of quality assessment and publication bias

**Figure 2:**
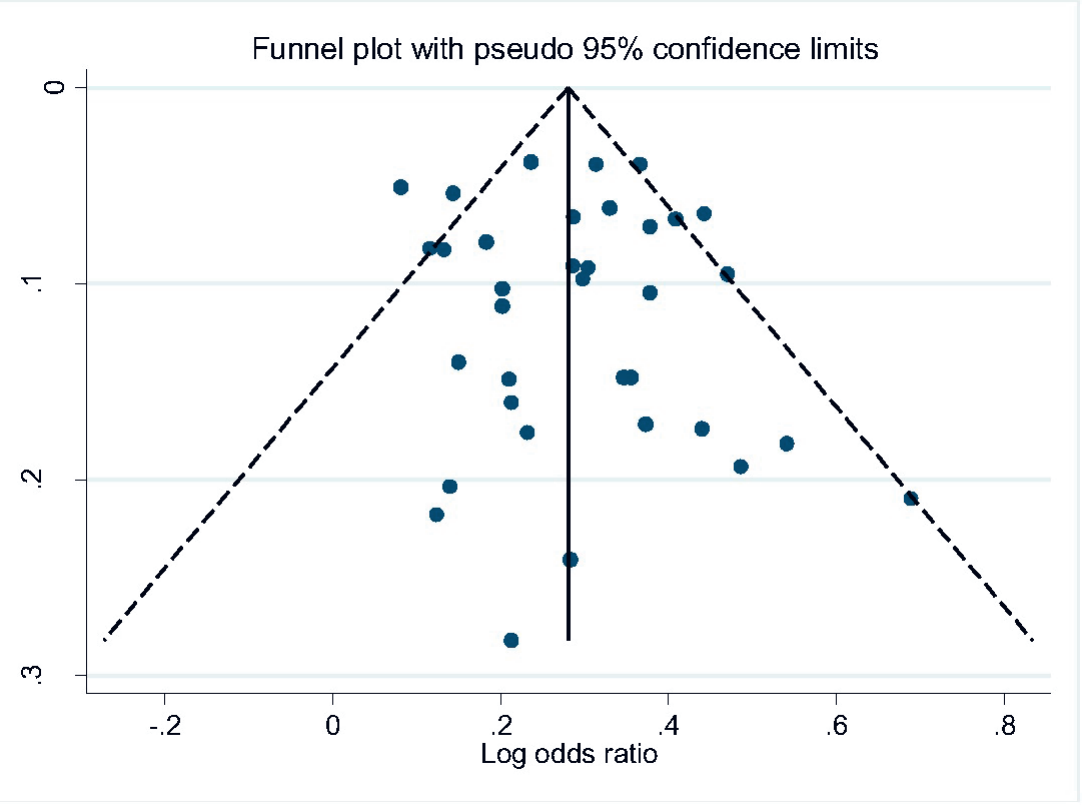
Funnel plot showing symmetrical distribution of selected studies indicating an absence of publication bias.

All studies included in the review were classified as being of moderate to high quality with low risk of bias (**Table 1**). We also assessed publication bias using Egger’s test. The shape of funnel plot did not reveal evidence of obvious asymmetry (**Figure 2**). The estimated bias coefficient was 0.151 with a standard error of 0.459. We also provided statistical evidence of the funnel plot symmetry using the Egger’s test, which did not reveal any evidence of publication bias or other small study effects (*P* = 0.601 for all comparisons).

#### Prevalence of hypertension

**Figure 3:**
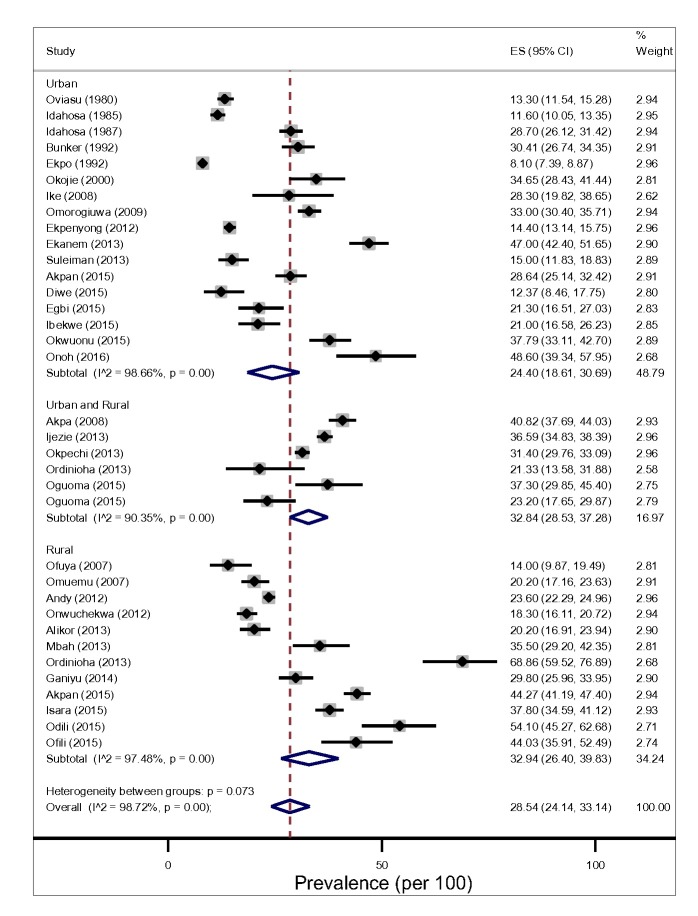
Pooled prevalence of hypertension among adults in the Niger delta settings.

The prevalence of hypertension in Niger Delta varied significantly across States and study settings (**Figure 3** and **Table 1**). Based on prevalence rates reported by individual studies using 140/90mmHg diagnostic measure, we found that the lowest and highest prevalence of hypertension was recorded in studies conducted in Cross River and River State each having a prevalence of 8.10% and 68.90 respectively [16,33]. In addition, the two studies were conducted more than a decade apart (1992-2013) and in the rural [17) and urban settings [57]. In contrast, Idahosa [28] reported the least prevalence of hypertension between the two studies with 160/95 and 160/100mmHg diagnostic measures. Another site with the highest prevalence rates of hypertension was also found Imo States. It reported a prevalence rate of 54.10% in 2015 and participant’s mean-age of 54.7 ± 16.3 [42,49].

From the random-effects meta-analysis, we estimated the prevalence of hypertension, and 95% CIs yielded 28.20% (95% CI 24.14-33.14). The *I*^2^ statistics was 97.40%, indicating statistically significant heterogeneity among the studies (**Figure 3**). There was no evidence of funnel plot asymmetry suggesting no evidence of publication bias (**Figure 2**). We compared the pooled prevalence estimates of hypertension based on two different hypertension cut-offs. We found that the pooled prevalence of hypertension for studies utilizing the 140/90mmHg measure (32 studies) only was significantly higher than the current estimates which provided pooled estimates for both 140/90 and 160/95 and 160/100 mm Hg measures (34 studies), 29.85% (95% CI 25.91-33.94).vs 28.20% (95% CI 24.14-32.45), *P* < 0.037. This suggests that 160/95 and 160/100 mm Hg cut-off point may have underestimated hypertension prevalence significantly.

### Pooled prevalence of hypertension by different study level characteristics

**Figure 4:**
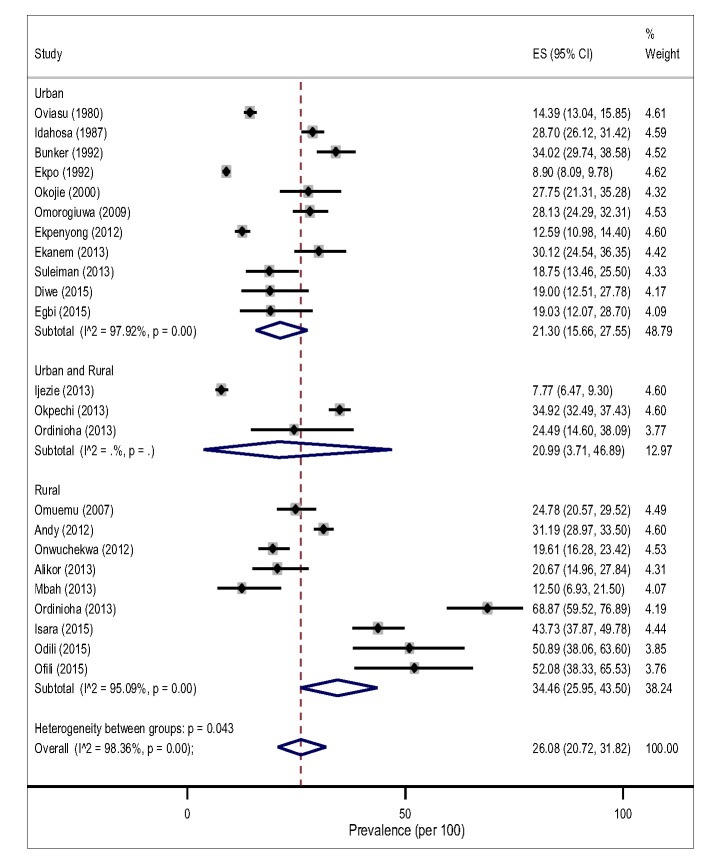
Pooled prevalence of hypertension among adult males in the Niger delta.

**Figure 5:**
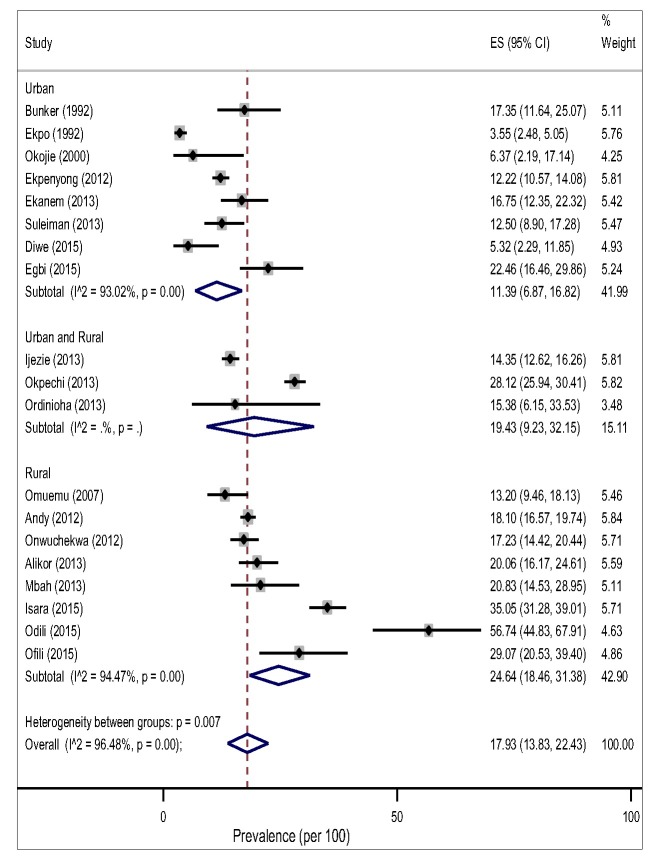
Pooled prevalence of hypertension among adult females in the Niger delta.

The pooled prevalence of hypertension tended to be higher in rural, 32.94% (95% CI 26.40-39.83, I^2^ = 97.48%) than in the urban settings, 24.40% (95% CI 18.61-30.69, I^2^ = 98.66%) However, this difference did not reach a statistical significance level (*P* = 0.183) for both gender (**Figure 3**).The result of the pooled estimates also found that men have a significantly higher prevalence compared to women, 26.08% (95% CI 20.72-31.82) vs 17.93% (95% CI 13.83-22.43), *P* = 0.000. Across settings, the pooled prevalence in the rural areas were significantly higher that urban areas for both gender (**Figure 4** and **Figure 5**).

**Figure 6:**
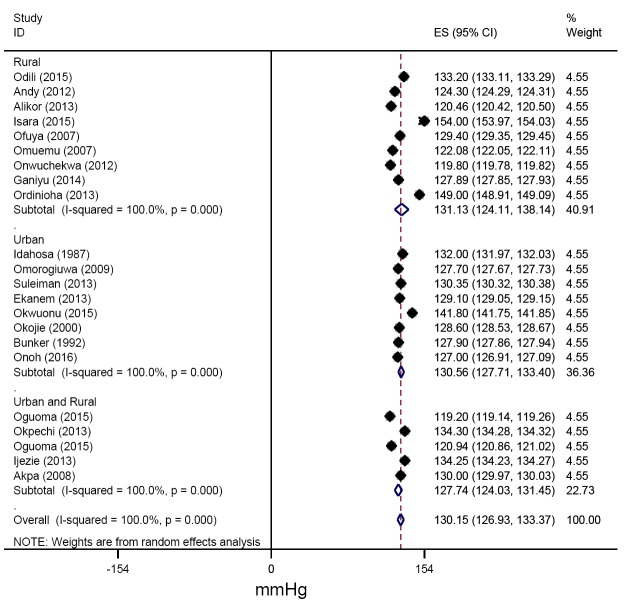
Pooled systolic blood pressure among adults in the Niger delta.

**Figure 7:**
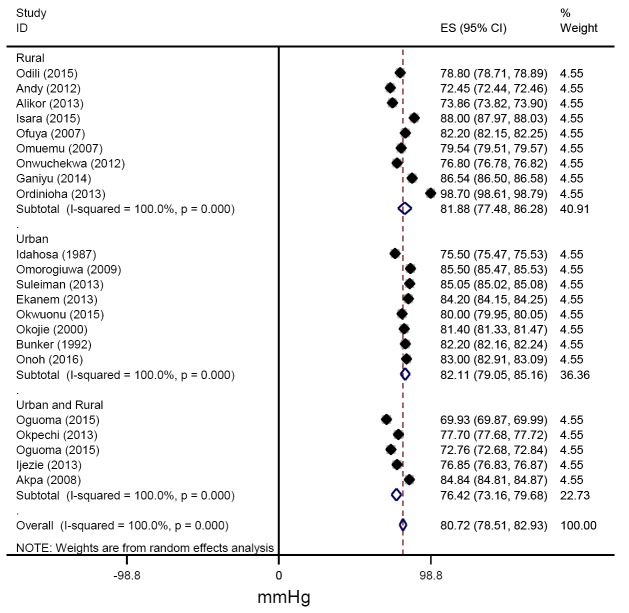
Pooled diastolic blood pressure among adults in the Niger delta.

From all studies reporting mean blood pressures, the pooled estimates of SBP across urban and rural settings were 130.56 (127.41-133.71) mmHg and 131.13(122.55-139.70) mmHg respectively (**Figure 6**). Similarly, the pooled estimates of DBP for urban, rural and for mixed settings were 82.11 (79.12-85.09), 81.88 (76.80-86.95) and 76.42 (73.13-79.71 mm Hg respectively (**Figure 7**).

We also carried a subgroup analysis to estimate the prevalence of hypertension by different risk factors (eg, age, gender, smokers, BMI, and alcohol use) in the Niger Delta (**Table 2**). We found that the prevalence of hypertension appeared to be higher among current and ex-smokers, those 65 years, over, alcohol drinkers, and among obese/overweight participants compared to their counterparts (**Table 2**).

**Table 2:** Prevalence of hypertension across several risk factors in the Niger Delta

Subgroup	Total Population (N)	Prevalence (95% CI)
**States:**		
Abia State	6264	34.21 (30.33-38.21)
Akwa Ibom State	4792	32.71 (16.01-52.05)
Bayelsa State	631	17.20 (14.34-20.25)
Cross River State	9069	13.89 (13.18-14.61)
Delta State	1232	30.41 (23.25-38.08)
Edo State	7224	25.47 (18.03-33.72)
Imo State	623	36.37 (17.93-57.14)
Rivers State	2880	29.29 (17.64-42.48)
**Age group (years):**		
15-44	5038	17.94 (14.04-22.19)
45-64	19139	38.10 (28.93-47.70)
65 and over	8538	47.26 (35.72-58.93)
**Body Mass Index (BMI):**		
Underweight	2506	10.72 (8.55-22.35)
Normal	2612	27.44 (18.65-37.18)
Overweight	2053	41.27 (25.11-58.43)
Obese	4084	44.13 (33.92-54.57)
**Smoking status:**		
Non-smoker	2156	18.07 (15.79-20.60)
Current smokers/ex-smokers	2828	26.49 (18.26-50.11)
**Alcohol use:**		
Non-drinkers	1637	21.55 (18.73-24.51)
Drinkers	3209	26.54 (22.21-38.35
**Intervals:**		
Pre-1989	3838	17.27 (8.60-18.17
1990-2009	8957	25.25 (14.09-35.21)
2010-2016	19930	30.53 (26.04-38.44)
**Study setting:**		
Urban	16481	24.40 (18.61-30.69)
Rural	9122	32.94 (26.40-39.83)
Urban/rural (mixed)	7112	32.84 (28.53-37.28)

### Factors modifying hypertension estimates and secular trend

**Figure 8:**
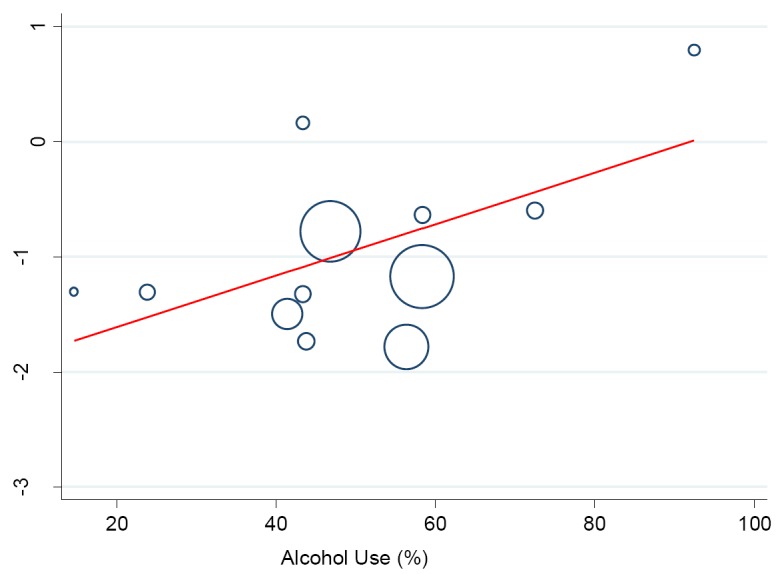
Meta-regression analysis showing the relationship between prevalence of hypertension and participants’ alcohol use. The size of the bubble corresponds to the study sample size.

A meta-regression analysis was performed to investigate the potential influence of lifestyle and sociodemographic factors on between-study heterogeneity in the prevalence of hypertension. We found a logarithm of hypertension prevalence rose by a factor of 0.02 with an increase in the proportion of drinkers/alcohol consumption (95% CI 0.00-0.05, *P* < 0.046) **(****Figure 8**). Similarly, a statistically significant effect was observed between hypertension prevalence which rose by a factor of 0.04 in the proportion of smokers/ex-smokers (95% CI 0.03-0.12), *P* < 0.016.

**Figure 9:**
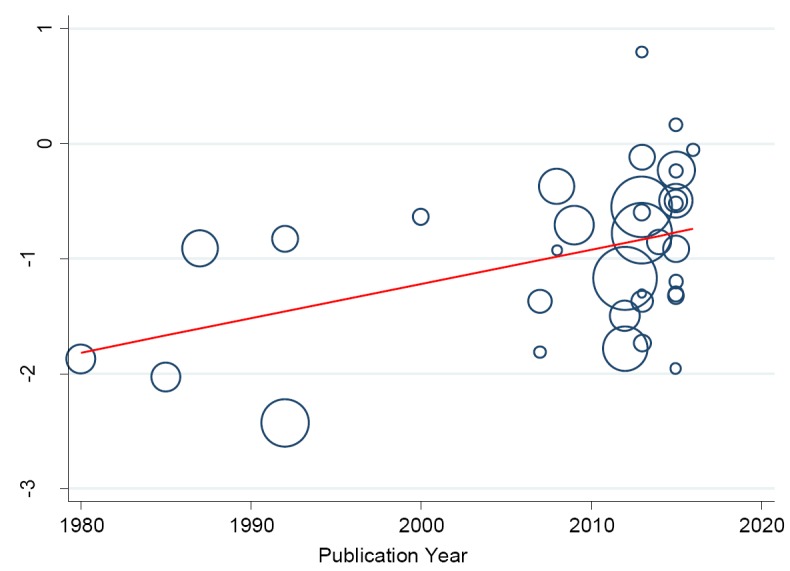
Meta-regression analysis showing the relationship between prevalence of hypertension and study publication year. The size of the bubble corresponds to the study sample size.

**Figure 10:**
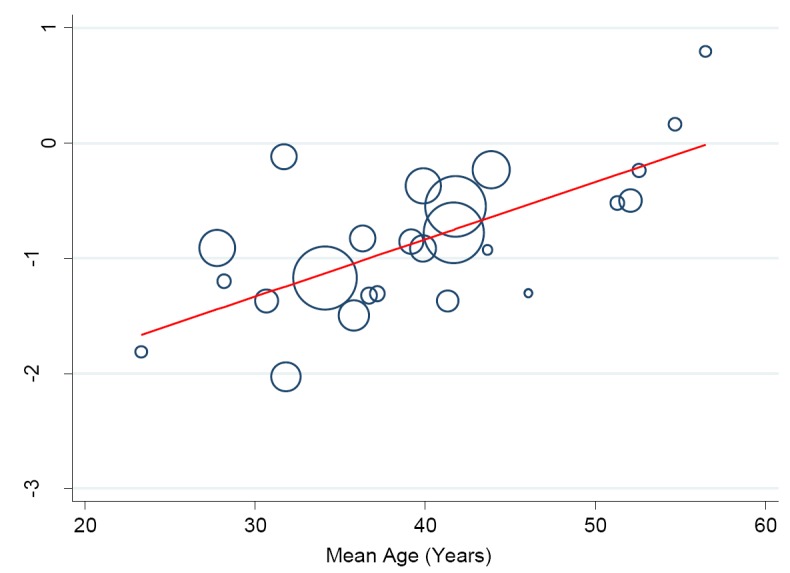
Meta-regression analysis showing the relationship between prevalence of hypertension and participants’ mean age. The size of the bubble corresponds to the study sample size.

In a separate analysis, we found some changes in the prevalence of hypertension in the Niger Delta over the past three decades. Specifically, from 1980 to 2016, we observed a continuous increase in prevalence of hypertension in the region (trend = 0.139, *P* = 0.0001) (**Figure 9**) such that the prevalence estimates have been increasing by 10.43% (5.73-15.14) every 10 years increase in participants mean-age (**Figure 10**).

## DISCUSSION

To our knowledge, this is the first and most comprehensive review to date that attempted to present the burden of hypertension in the Niger Delta or any region with significant oil and gas production activities. We presented the pooled analysis of the prevalence of hypertension among vulnerable adults’ populations living in a hugely environmentally polluted region. Our study corroborates the notion that hypertension is already a major public health burden in this population. We collated data on 32715 adults in the Niger Delta with an estimated population of 32 million people. We estimated an overall crude hypertension prevalence of 28.20%. This result is higher than previous estimates of 18.4% [11]. However, the result is consistent with the recent review estimate of 28.9% [10] for the whole country. The result is lower than the finding of the national survey that found a prevalence of 44.9% among adults age ≥40 years and mean age 55.9 ± 12.4 years [57].

Interestingly, our rural-urban estimates of 32.0% vs 24.07% were significantly different from 15.0% vs 22.30% reported in 2012 [11], and the most recent estimates of 26.40% vs 30.60% [10]. This is also a huge contrast to the national survey estimates of 43.0% vs 51.6% among older adults of ≥40 years [57]. Our result is also higher than the prevalence estimates in many countries and population groups in sub-Saharan Africa suggesting an interplay of both epidemiological, demographic and in recent times, environmental transitions [58,59]. This may also be due to nutritional changes and potentially better surveillance strategy that increases the detection of hypertensive cases [10]. Our study also noted that across all the States where the data for the study were available, we found that the mean-age of the participants was 38.43 ± 2.0 years, which is about a decade younger than the previous review that reported lower hypertension prevalence for the whole country [10].

We found a high preponderance of several risk factors for hypertension. In particular, ageing and excess body weight and being a current/ex-smoker and drinker were highlighted. All these have widely been reviewed previously [11]. The pattern of age-specific increase in hypertension prevalence is clearly marked within three age brackets for both genders. We found a significant difference among the highest proportion of hypertensives aged 65 years and over (47.26%) compared to those aged 15-44 years (17.94%) or 45-64 years (38.10*%).* This is consistent with the fact that age is an independent risk factor for hypertension.

Substantial inter-State and study sites variation in the prevalence of hypertension was found, although this may be linked to fewer data points, we reported that the pooled estimate for hypertension in rural areas appeared to be higher but was not statistically significant. This non-significant difference could not be related to the difference in mean-age. We noted previously that there was no significant difference in mean-age of participants in rural compared to urban settings, *P* = 0.45. Other possible explanations could be differences in lifestyle factors, high poverty level (socioeconomic factors), and investment in health care services, and most importantly the increased exposure to oil and gas-pollution in the rural host-communities [60].

Recent reviews in Nigeria are consistent with the urban-rural divide with the urban population having a higher prevalence of hypertension [10]. This is largely explained by not only urbanisation and adoption of western diet (particularly food rich in salt and saturated fat) as a result of nutritional transitions currently occurring in Nigeria and other lower-income countries but also access and availability of health care and socioeconomic factors [59]. Sedentary lifestyle and engagement in jobs with minimal physical activities are also common occurrences among urban dwellers. However, it has been reported that rural host-communities were also exposed to western lifestyle due to the influence of oil and gas workers in their neighbourhood [61].

The non-significant difference between the rural and urban areas could also be related to the reverse rural-urban migration reported when urban dwellers find it difficult to[3] cope with the economic situation and vulnerability related to urban life, or when senior citizen retires from active service and prefers to return to natural resource-rich rural settlements [62-64]. Although it is difficult to establish the risk level associated with exposure to pollution occurring because of quasi-refining operations and other unregulated activities, we cannot discountenance the positive influence of socioeconomic factors to the observed outcome. This needs to be investigated further particularly in the Niger Delta rural oil and gas host communities.

### Policy implication

Understanding and addressing the current prevalence of hypertension in this population is important for reducing disparities in health. The health priorities of many low- and middle-income countries particularly in sub-Saharan Africa and south Asian countries have remained infectious diseases mainly HIV/AIDS, Malaria and Tuberculosis. This is in addition to high poverty, malnutrition, illiteracy, and social discrimination [10]. The socio-economic impact of increased hypertension prevalence in this population would mean increased loss in productivity and reduced investment both in health and other areas [11]. The increase in hypertension prevalence by 10.43% (5.73-15.14), every 10 years in the Niger Delta is huge and may suggest that the rates of cardiovascular and metabolic complications such as cerebrovascular accidents, heart failure, and renal failure are likely to increase in the coming years.

With the finding of significant increase in the prevalence of hypertension in the Niger Delta, options for routine surveillance and cost-effective prevention of major risk factors such as lifestyle factors [4] remain important public health policy priority. This is important particularly in the oil and gas host-communities where the care for conditions like hypertension and the standard of health service delivery is generally inadequate [3,45]. Beside high cost of medication and health care seeking behaviour with individual preferring low-cost sub-standard health facilities[44,54], the detection and overall management are poor in rural areas in comparison to urban population [10]. As a result of the double burden of communicable and non-communicable diseases in Nigeria, it appears that the high estimates found in this study will continue in an upward trend. Unfortunately, the upward trend will lead to increased comorbidity and fatality particulalry due to public health policy focusing on the prevention and control of infectious diseases (such as HIV/AIDS, Poliomyelitis, Malaria and Tuberculosis) including maternal, perinatal and nutrition-related conditions.

### Strengths and limitations

The overall strengths and limitations of this meta-analysis warrant careful consideration. We conducted a comprehensive search of databases to ensure that all relevant articles were identified. We also reduced potential bias in the conduct of this review by having the authors independently scan through the search output and extract relevant data. This was in addition to a detailed quality assessment and publication bias, with eligible studies having no small study effects. In addition, we included only community-based studies that constitute the best way to determine the true prevalence of hypertension and systematically identified the possible sources of heterogeneity using meta-regression analysis. This is in addition to the estimate of hypertension trend in the region.

Despite the strength of the review, some limitations in the present study deserve attention. We did not provide data on environmental pollution in the Niger Delta data. Although such data are important for environmental management and health care planning, such estimates may be unreliable due to conflicting information from stakeholders particularly the oil and gas companies that leverage on weak environmental regulation and corruption to evade proper accountability and persecution [65]. There may be a risk of potential bias because most of the elevated blood pressure reading could not be confirmed as diagnosed hypertension. In addition, we cannot disregard the probability that health outcomes such as hypertension may influence reports of smoking, drinking habits and other lifestyle factors, and not vice versa [66]. Similarly, lack of information on physical activity, ethnicity, biomarkers, pollutants monitoring and characterisation may have limited our understanding of the influence of these to increased hypertension estimates and its aetiology.

## CONCLUSIONS

In conclusion, our study provides an up-to-date estimate that reflects the huge prevalence of hypertension in Niger Delta. The review provided interesting learning in view of the significantly higher burden of hypertension recorded in the region. The study found that the pooled estimates of studies conducted in the rural population tended to be higher compared to the urban residents, however, the difference did not reach a statistical significance level. These findings therefore should be treated with caution in view of the previously reported national surveys that found different results [57,67]. The slight difference may be due to fewer data points as seen in the included studies. These need to be further tested in longitudinal studies with large sample size. The current prevalence of hypertension is the Niger Delta is very heterogeneous in terms of the overall estimates and risk factors, however, the influence of unmeasured factors such as diet types, reverse migration and other extrinsic factors particularly environmental pollution that has been implicated in huge socio-economic inequality and other co-morbid conditions cannot be ruled out.
